# Transitioning From Blind to Real‐Time Ultrasound‐Guided Kidney Biopsy in Children: Diagnostic Yield and Bleeding Outcomes in a Vietnamese Tertiary Referral Hospital, a Retrospective Cohort Study

**DOI:** 10.1155/ijne/7390086

**Published:** 2026-07-30

**Authors:** Binh Thanh Le, Tien Quoc Nguyen, Anh Xuan Nguyen, Cam Thao Nguyen, Tru Huy Vu, Dinh Quang Truong, Tien Minh Nguyen, Quang Duc Nguyen, Linh Ngoc Huynh, Khanh Ngoc Minh Nguyen, Tam Ngoc Minh Bui, Siyu Chen, Binbin Ji, Jackson Tan, Minh Cuong Duong

**Affiliations:** ^1^ Department of Nephro-endocrinology, City Children’s Hospital, Ho Chi Minh City 700000, Vietnam; ^2^ Pediatrics Department, Tam Anh Hospital, Ho Chi Minh City 700000, Vietnam; ^3^ City Children’s Hospital, Ho Chi Minh City 700000, Vietnam; ^4^ Department of Nephro-endocrinology, Children’s Hospital 1, Ho Chi Minh City 700000, Vietnam, nhidong.org.vn; ^5^ Department of Pathology and Forensic Medicine, Pham Ngoc Thach University of Medicine, Ho Chi Minh City 700000, Vietnam, pnt.edu.vn; ^6^ Department of Radiology, City Children’s Hospital, Ho Chi Minh City 700000, Vietnam; ^7^ Health Screening Department, University Medical Center Ho Chi Minh City, 215 Hong Bang Street Cho Lon Ward, Ho Chi Minh City 700000, Vietnam, bvdaihoc.com.vn; ^8^ Centre for Health Behaviours Research, JC School of Public Health and Primary Care, Faculty of Medicine, The Chinese University of Hong Kong, Hong Kong, China, cuhk.edu.hk; ^9^ School of Nursing, Hunan University of Chinese Medicine, 300 Xueshi Road Yuelu District, Changsha 410208, China, hnctcm.edu.cn; ^10^ PAPRSB Institute of Health Sciences, Universiti Brunei Darussalam, Gadong, Bandar Seri Begawan BE1410, Brunei Darussalam, ubd.edu.bn; ^11^ School of Population Health, University of New South Wales, Building C29 HTH Level 5, Kensington 2033, New South Wales, Australia, unsw.edu.au

**Keywords:** biopsy complications, blind biopsy, glomerular yield, paediatric kidney biopsy, ultrasound-guided biopsy

## Abstract

**Background:**

Kidney biopsy is the gold standard for diagnosing renal parenchymal diseases, guiding treatment and assessing prognosis. Real‐time ultrasound‐guided biopsy is widely regarded as the preferred technique. However, the implementation may be affected by equipment availability, trained personnel and workflow constraints in resource‐limited settings worldwide.

**Objective:**

This study compared the safety and effectiveness of real‐time ultrasound‐guided and blind kidney biopsy techniques in a paediatric tertiary referral hospital in Vietnam and examined the practical implications of transitioning to real‐time ultrasound guidance.

**Methods:**

A retrospective cohort study was conducted at the Nephro‐Endocrinology Department, City Children’s Hospital, Ho Chi Minh City, Vietnam, from May 2018 to April 2025. All native kidney biopsies performed during the study period were included. A standardised questionnaire was used to extract data from medical records, including demographics, biopsy techniques, glomerular yield, samples containing ≥ 10 and ≥ 25 glomeruli, and inadequate yield and complications, including macroscopic haematuria and major complications.

**Results:**

Among 221 participants (median age: 11 [IQR 7–13] years), 173 underwent real‐time ultrasound‐guided biopsy and 48 used the blind technique. The ultrasound‐guided group had a significantly higher median glomerular count (*p* = 0.01) and greater proportion of samples with ≥ 10 (*p* < 0.001) and ≥ 25 glomeruli (*p* = 0.04). There were no significant differences in inadequate sample rates (*p* = 0.06), macroscopic haematuria (*p* = 0.63) or major complications (*p* = 0.60). Lower eGFR was independently associated with post‐biopsy bleeding events, mainly macroscopic haematuria (AOR = 0.809 per 10 mL/min/1.73 m^2^ increase, 95% CI 0.671–0.975, *p* = 0.03).

**Conclusion:**

In this Vietnamese paediatric tertiary referral setting, real‐time ultrasound‐guided biopsy provided superior tissue adequacy compared with the blind technique, without a detectable increase in macroscopic haematuria or major complications. Lower estimated glomerular filtration rate was associated with post‐biopsy bleeding events in this cohort, mostly macroscopic haematuria rather than major bleeding.

## 1. Introduction

Percutaneous kidney biopsy is the gold standard for diagnosing renal parenchymal diseases, guiding therapy and assessing prognosis [1]. In developing countries, where glomerulonephritis remains an important contributor to chronic kidney disease, access to a high‐quality and safe kidney biopsy service is particularly relevant for diagnostic confirmation and treatment planning [2]. Adequate tissue sampling is essential, particularly for detecting focal lesions [3, 4]. While 10–20 glomeruli are generally recommended for light microscopy (LM) [1, 3, 5], at least 20 glomeruli are advised to minimise misdiagnosis [3] and ≥ 25 glomeruli provide approximately 95% sensitivity for detecting focal lesions [4]. Given its invasive nature, bleeding remains the most concerning complication [6].

Kidney biopsy using an automated biopsy gun with ultrasound guidance is now a standard practice, performed either as real‐time ultrasound‐guided biopsy or blind (pre‐marked) biopsy [6, 7]. Real‐time ultrasound‐guided biopsy is widely preferred for its higher glomerular yield and lower complication rate [1, 5], and has become the standard of care for many centres [1]. Nevertheless, blind biopsy remains an optional technique in some guidelines [7] and may still be used in resource‐limited or transitioning healthcare settings where real‐time ultrasound support, trained operators or procedural coordination may not be consistently available [8, 9]. Most comparative studies have focused on adults, and some have reported no differences between techniques in sample adequacy or complication rates [1]. In children, prior studies have supported the usefulness and safety of ultrasound guidance, but comparative paediatric data remain limited, particularly from low‐ and middle‐income settings [8, 10]. At our institution, blind biopsy was used before February 2020, after which real‐time ultrasound‐guided biopsy was implemented through collaboration between nephrologists and radiologists. This transition provided an opportunity to evaluate whether adopting real‐time ultrasound guidance improved diagnostic yield without increasing post‐biopsy complications in a Vietnamese paediatric tertiary referral hospital. This study aimed to compare the safety and effectiveness of real‐time ultrasound‐guided and blind biopsy techniques in children by evaluating glomerular yield, sample adequacy, macroscopic haematuria and major complications, while considering the practical relevance of implementing real‐time ultrasound guidance in our healthcare setting.

## 2. Methods

### 2.1. Study Design, Context and Participants

A retrospective cohort study was conducted at the Nephro‐Endocrinology Department of City Children’s Hospital (CCH) in Ho Chi Minh City, Vietnam, from May 1, 2018 to April 30, 2025. CCH is among the largest tertiary referral hospitals for paediatric patients in southern Vietnam. Standardised paper‐based medical records were used until May 1, 2023, after which standardised, electronic records were implemented. The Nephro‐Endocrinology Department manages paediatric patients with various kidney diseases, while surgical cases are referred to the Urology Department. The study was approved by the CCH Ethics Committee (approval No. 1010/QĐ‐BVNDTP). Informed consent was not required as only de‐identified medical record data were analysed.

All patients who underwent native kidney biopsy during the study period were included. Biopsy indications were: (1) steroid‐resistant nephrotic syndrome (SRNS); (2) other indications for nephrotic syndrome (patients with age less than a year, serum complement decrease, hepatitis B infection or macroscopic haematuria); (3) before calcineurin inhibitor treatment; (4) rapid progressive glomerulonephritis (RPGN); (5) unknown decrease in estimated glomerular filtration rate (eGFR); (6) recurrent macroscopic haematuria; (7) persistent significant proteinuria (proteinuria > 0.5 mg/m^2^/day) with or without haematuria; (8) nephrotic‐nephritic syndrome; (9) kidney involvement due to systemic diseases (including lupus nephritis, IgA vasculitis) and (10) follow‐up biopsy [1, 3]. Indications were determined by treating physicians and reviewed by nephrologists before biopsy. Kidney transplant biopsies were excluded.

Before February 2020, biopsies were performed using the blind technique. Subsequently, the real‐time ultrasound‐guided biopsy was adopted. All biopsies were performed by nephrologists, with a radiologist assisting in skin marking (blind technique) or in probe handling (real‐time ultrasound‐guided biopsy).

### 2.2. Kidney Biopsy Preparation and Techniques

#### 2.2.1. Preparation

Bleeding risk factors, including uncontrolled hypertension, thrombocytopenia, prothrombin time and severe anaemia, were corrected before biopsy [6]. Medications affecting coagulation, including nonsteroidal anti‐inflammatory drugs and antiplatelet agents, were discontinued 1 week prior to the biopsies [3]. Anticoagulants were withheld as per the guidelines [3].

#### 2.2.2. Procedure

Kidney biopsy was performed with a 16‐gauge automated biopsy gun (C. R. Bard, Inc., Murray Hill, NJ, USA) under intravenous sedation in the procedure room.

Blind technique was performed in the prone position. A radiologist identified the kidney lower pole and depth via ultrasound and marked the biopsy site. After povidone‐iodine skin disinfection and local anaesthesia with 2% lidocaine, a small incision was made, and the biopsy needle was inserted to the target depth that was confirmed when the tip of the proper needle passed the kidney capsule and by respiratory synchronising movement of the proper needle. Samples were collected during expiration and placed in 10% buffered formalin.

Real‐time ultrasound‐guided biopsy was also performed in the prone position with the ultrasound probe in a sterile cover containing ultrasound gel. The radiologist stabilised the ultrasound probe, while the nephrologist advanced the biopsy needle under real‐time ultrasound guidance to get closer to the kidney capsule [1]. The remaining steps were identical to blind technique.

The number of needle passes per session was recorded. Biopsy was limited to three passes per session, regardless of adequacy [11]. Specimens were transported within 1 h to the Pathology Department of Children’s Hospital 1, another large tertiary hospital for children in southern Vietnam, for LM and immunofluorescence (IF) using paraffin‐embedded tissue [5]. All obtained glomeruli were analysed under LM. At Children’s Hospital 1, IF staining was performed on sections cut from paraffin‐embedded tissue instead of frozen tissue, as part of their routine IF protocols [12, 13]. Electron microscopy was unavailable.

### 2.3. Post‐Biopsy Follow‐Up

Patients were asked to do bed rest for 24 h, including the first 6 h in the supine position. Vital signs, including heart rate and blood pressure, were closely monitored as per the CCH’s protocol, and urine was collected on each void for macroscopic haematuria detection [5]. Kidney ultrasound and haematocrit were not performed routinely after biopsy, except for cases with persistent macroscopic haematuria or changes in vital signs as recommended [1, 7].

### 2.4. Outcome Parameters

The efficacy of biopsy techniques was evaluated based on the number of glomeruli per biopsy and the rate of inadequate tissue samples. The efficacy was also examined based on the frequencies of (1) tissue samples with at least 10 glomeruli, which is the suggested minimum number of glomeruli that should be obtained, and (2) tissue samples containing at least 25 glomeruli, which is an ideal number of obtained glomeruli [3, 4]. An inadequate tissue sample was defined as a biopsy specimen that, following discussion with histopathologists, failed to yield a conclusive pathological diagnosis [11]. The safety of biopsy procedures was evaluated based on macroscopic haematuria and major complications. Major complications were defined as death, perinephric abscess or any bleeding requiring erythrocyte transfusion or invasive post‐biopsy interventions, including surgery or embolization [1]. For predictor analysis, post‐biopsy bleeding events were defined as macroscopic haematuria or bleeding requiring erythrocyte transfusion or invasive post‐biopsy intervention.

### 2.5. Data Collection

A standardised questionnaire was used to retrieve information from the patients’ medical records. Data included demographics, clinical data (height, weight, blood pressure), laboratory results (serum creatinine, haemoglobin, platelet counts and prothrombin time‐INR), biopsy indication, biopsy techniques, and post‐biopsy complications (macroscopic haematuria, perinephric abscess, bleeding requiring erythrocyte transfusion or other intervention including surgery or embolization). Hypertension was defined based on the guidelines of the American Academy of Pediatrics for screening and management of high blood pressure in children and adolescents [14]. Body mass index (BMI) was calculated and obesity was defined when BMI was > 95^th^ percentile for age and sex, as recommended by the World Health Organization (WHO) [15]. The eGFR was calculated based on the chronic kidney disease in children and young adults under 25 years old (CKiD U25) equation, except children younger than 12 months of age, where the revised Schwartz equation was used [16, 17].

### 2.6. Statistical Analysis

SPSS version 26 (IBM Corp, Armonk, New York) was used to manage and analyse data. Categorical variables, which were presented as an absolute count and percentage, were compared using chi‐squared and Fisher’s exact tests. Continuous variables presented as median (interquartile range [IQR]; minimum–maximum) were compared using the Mann–Whitney *U* test. A multivariable logistic regression model was developed to identify predictors of post‐biopsy bleeding events and included all variables with a *p* value < 0.25 in univariable analysis and other variables that were clinically judged to be associated with these events [18]. Alpha was set at 5% level.

## 3. Results

### 3.1. Baseline Characteristics of Study Participants

**FIGURE 1:**
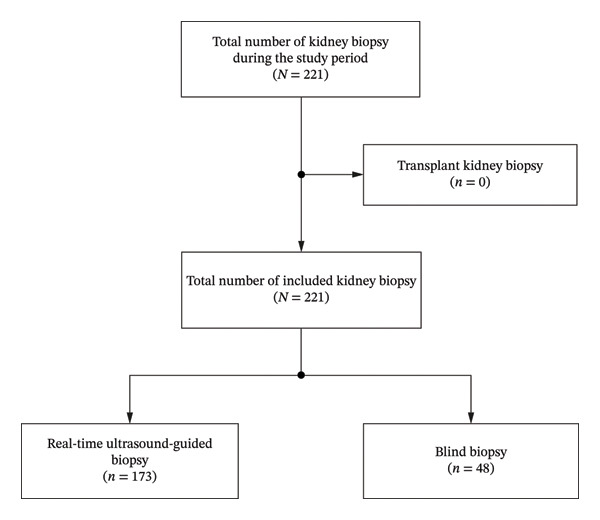
Flowchart of study participants.

A total of 221 patients underwent native kidney biopsy during the study period, including 48 blind biopsies and 173 real‐time ultrasound‐guided biopsies (Figure 1). Among these, 41.2% were male, and the median age was 11 years (IQR: 7–13; range: 0–15 years) (Table 1). Overall, 28.1% of patients had arterial hypertension and 20.8% were classified as obese. The median eGFR was 86 mL/min/1.73 m^2^ (IQR: 60–100; range: 6–170), and the median pre‐biopsy haemoglobin level was 11.5 g/dL (IQR: 9.4–13.4; range: 5.8–17.8). The median platelet count was 332 × 10^3^/mm^3^ (IQR: 229–429; range: 73–1,221), and the median prothrombin time‐INR was 0.98 (IQR: 0.92–1.04; range: 0.77–1.55). No significant differences were found in baseline characteristics between patients undergoing real‐time ultrasound‐guided and blind biopsies, except for pre‐biopsy haemoglobin levels and prothrombin time‐INR values.

**TABLE 1:** Baseline clinical and laboratory characteristics (*N* = 221).

Characteristics	Summary statistics^∗^			*p* value
	Total (*n* = 221)	Real‐time ultrasound‐guided biopsy (*n* = 173)	Blind biopsy (*n* = 48)	
Age (years)	11 (7–13; 0–15)	11 (8–14; 0–15)	9.5 (7–13; 0–15)	0.11^a^
Male	91 (41.2)	70 (40.5)	21 (43.8)	0.68^b^
Arterial hypertension	62 (28.1)	47 (27.2)	15 (31.2)	0.58^b^
Obesity	46 (20.8)	33 (19.1)	13 (27.1)	0.23^b^
eGFR (ml/min/1.73 m^2^)	86 (60–100; 6–170)	85 (62–97; 6–170)	91 (58–110; 12–132)	0.17^a^
Pre‐biopsy haemoglobin (g/dL)	11.5 (9.4–13.4; 5.8–17.8)	11.2 (9.0–13.1; 6–17.8)	12.2 (11.0–13.9; 5.8–17.3)	0.02^a^
Pre‐biopsy platelet count (x 1000/mm^3^)	332 (229–429; 73–1221)	325 (208–430; 73–1221)	337 (272–414; 88–842)	0.63^a^
Prothrombin time‐INR	0.98 (0.92–1.04; 0.77–1.55)	0.96 (0.90–1.03; 0.77–1.55)	1.02 (0.94–1.06; 0.82–1.29)	0.03^a^
Biopsy indications				
Lupus nephritis	92 (41.6)	80 (46.2)	12 (25)	0.01^b^
Steroid‐resistant nephrotic syndrome	55 (24.9)	47 (27.2)	8 (16.7)	0.14^b^
IgA vasculitis nephritis	16 (7.2)	12 (6.9)	4 (8.3)	0.74^c^
Before calcineurin inhibitor treatment	11 (5.0)	6 (3.5)	5 (10.4)	0.05^c^
Others^∗∗^	47 (21.3)	28 (16.2)	19 (39.6)	< 0.001^b^

Abbreviation: eGFR, estimated glomerular filtration rate.

^∗^Continuous variables are presented as median (IQR; min–max), while categorical variables are presented as *N* (%).

^∗∗^Including nephrotic–nephritic syndrome, unknown origin eGFR decrease, rapid progressive glomerulonephritis, recurrent macroscopic haematuria, persistent significant proteinuria with/without haematuria, other indications for nephrotic syndrome (age less than 1 year, serum complement decrease, hepatitis B infection or macroscopic haematuria) and follow‐up biopsy.

^a^Mann–Whitney *U* test.

^b^Chi‐squared test.

^c^Fisher’s exact test.

Lupus nephritis (41.6%), SRNS (24.9%) and IgA vasculitis nephritis (7.2%) were the most frequent biopsy indications. The proportion of patients with lupus nephritis was significantly higher in the real‐time ultrasound‐guided biopsy group compared with the blind biopsy group (*p* = 0.01). Patients with lupus nephritis also had significantly lower median pre‐biopsy haemoglobin levels than those with other indications (*p* < 0.001; data not shown). There were no significant differences in prothrombin time‐INR values between most biopsy indication groups, except patients with SRNS, who had significantly lower INR values compared to the “others” indication group (*p* = 0.027; data not shown).

### 3.2. Efficacy of Biopsy Techniques

There was no significant difference in the median number of needle passes between the two biopsy technique groups (*p* = 0.58) (Table 2). Only one patient required four passes in a single biopsy, and this occurred in the real‐time ultrasound‐guided biopsy group. This case underwent a safety evaluation before the fourth pass and experienced no post‐biopsy complications (data not shown).

**TABLE 2:** Kidney biopsy results by biopsy techniques (*N* = 221).

Characteristics	Summary statistics^∗^			*p* value
	Total (*n* = 221)	Real‐time ultrasound‐guided biopsy (*n* = 173)	Blind biopsy (*n* = 48)	
Number of needle passes	2 (2–2; 1–4)	2 (2–2; 1–4)	2 (2–3; 1–3)	0.58^a^
Number of core samples	2 (2–2; 1–3)	2 (2–2; 1–3)	2 (1–2; 1–3)	< 0.001^a^
Number of glomeruli per biopsy	24 (19–35; 4–56)	25 (20–35; 5–56)	22 (16–31; 4–49)	0.01^a^
Tissue samples with ≥ 10 glomeruli	208 (94.1)	168 (97.1)	40 (83.3)	< 0.001^b^
Tissue samples with ≥ 25 glomeruli	107 (48.4)	90 (52.0)	17 (35.4)	0.04^c^
Inadequate tissue samples	1 (0.5)	0 (0.0)	1 (2.1)	0.06^b^

^∗^Continuous variables are presented as median (IQR; min–max), while categorical variables are presented as *N* (%).

^a^Mann–Whitney *U* test.

^b^Fisher’s exact test.

^c^Chi‐squared test.

In contrast, the number of core samples (*p* < 0.001) and glomeruli per biopsy (*p* = 0.01) were significantly higher in the real‐time ultrasound‐guided biopsy group than in the blind biopsy group. Similarly, the proportion of specimens containing ≥ 10 glomeruli (*p* < 0.001) and ≥ 25 glomeruli (*p* = 0.04) was significantly greater in the real‐time ultrasound‐guided biopsy group. Only one inadequate biopsy was recorded, consisting of four glomeruli without histopathologic findings, and this occurred in the blind biopsy group (data not shown).

### 3.3. Safety of the Biopsy Procedures

Among all participants, macroscopic haematuria occurred in 6.8% (15/221), whereas erythrocyte transfusion due to symptomatic perinephric haematoma was required in 0.5% (1/221) (Table 3). There were no statistically significant differences between the two biopsy technique groups for either macroscopic haematuria or major complications (*p* > 0.05). No additional major complications were reported.

**TABLE 3:** Comparison of post‐biopsy complications by biopsy techniques (*N* = 221).

Characteristics	Summary statistics, N (%)			*p* value^a^
	Total (*n* = 221)	Real‐time ultrasound‐guided biopsy (*n* = 173)	Blind biopsy (*n* = 48)	
Macroscopic haematuria	15 (6.8)	11 (6.4)	4 (8.3)	0.63
Major complications				
Symptomatic perinephric haematoma with erythrocyte transfusion required	1 (0.5)	1 (0.6)	0 (0)	0.60
Perinephric abscess	0	0	0	
Invasive post‐biopsy intervention or surgery	0	0	0	
Deaths	0	0	0	

^a^Fisher’s exact test.

### 3.4. Predictors of Post‐Biopsy Bleeding Events

Lower eGFR was the only significant predictor of post‐biopsy bleeding events (AOR = 0.809 per 10 mL/min/1.73 m^2^ increase, 95% CI 0.671–0.975, *p* = 0.03) (Table 4). Because only one patient required erythrocyte transfusion, the study was not powered to evaluate predictors of major bleeding.

**TABLE 4:** Univariable analysis and multivariable logistic regression model for predictors of post‐biopsy bleeding events (*N* = 221).

Variables	Univariable analysis^∗^				Multivariable analysis	
	Post‐biopsy bleeding events		Or (95% CI)	*p* value	Adjusted OR (95% CI)	*p* value
	Yes (*n* = 16)	No (*n* = 205)				
Age, years	10.5 (7–12; 0–15)	11 (7–13; 0–15)	—	0.51^a^	—	—
Male	7 (43.8)	84 (41.0)	1.120 (0.402–3.126)	0.83^b^	—	—
Arterial hypertension	2 (12.5)	60 (29.3)	0.345 (0.076–1.566)	0.15^c^	0.235 (0.046–1.205)	0.08
Obesity	2 (12.5)	44 (21.5)	0.523 (0.114–2.387)	0.40^c^	0.589 (0.119–2.915)	0.52
eGFR (mL/min/1.73 m^2^)	64 (39–94; 12–118)	86 (63–101; 6–170)	—	0.09^a^	0.809 (0.671–0.975)^d^	0.03
Pre‐biopsy haemoglobin (1 g/dL)	11.8 (10.8–13.6; 7.1–16.5)	11.5 (9.2–13.3; 5.8–17.8)	—	0.41^a^	1.187 (0.942–1.496)^e^	0.15
Pre‐biopsy platelet count (1000/mm^3^)	311 (214–387; 103–481)	335 (231–430; 73–1221)	—	0.44^a^	0.970 (0.930–1.013)^f^	0.17
Prothrombin time‐INR	0.95 (0.92–1.08; 0.84–1.13)	0.98 (0.92–1.04; 0.77–1.55)	—	0.71^a^	—	—
Number of needle passes (1 pass)	2 (2‐2; 2‐3)	2 (2–2; 1–4)	—	0.90^a^	1.241 (0.412–3.733)^g^	0.70
Real‐time ultrasound‐guided biopsy	12 (75.0)	161 (78.5)	0.820 (0.252–2.667)	0.74^c^	—	—

^∗^Continuous variables are presented as median (IQR; min–max), while categorical variables are presented as N(%).

^a^Mann–Whitney *U* test.

^b^Chi‐squared test.

^c^Fisher’s exact test.

^d^for every 10 mL/min/1.73 m^2^ increase in pre‐biopsy eGFR.

^e^for every 1 g/dL increase in pre‐biopsy haemoglobin levels.

^f^for every 10,000/mm^3^ increase in pre‐biopsy platelet count.

^g^for every additional needle pass.

## 4. Discussion

Our study demonstrated that, in a paediatric tertiary referral hospital in Vietnam, real‐time ultrasound‐guided biopsy provides superior tissue adequacy compared with the blind technique, without a detectable increase in macroscopic haematuria or major complications. All participants’ pre‐biopsy screening results were within the recommended safety ranges for a low‐risk biopsy procedure [19]. Baseline characteristics, including bleeding risk tests, were similar between groups, except for pre‐biopsy haemoglobin and prothrombin‐INR. The higher prothrombin‐INR in the blind group was likely due to a greater proportion of patients with ‘others’ biopsy indications, who had higher median values. Lower haemoglobin levels in the real‐time ultrasound‐guided biopsy group were likely explained by the higher prevalence of lupus nephritis, which is commonly associated with anaemia [20].

Real‐time ultrasound guidance is widely regarded as the preferred approach for paediatric kidney biopsy, and its technical advantages over blind biopsy have been reported in prior paediatric studies [8, 10]. However, implementation may still vary across healthcare settings because real‐time ultrasound‐guided biopsy requires appropriate equipment, trained personnel, procedural coordination and sustained operator experience [1, 7, 11, 19]. The present study therefore adds local implementation evidence from a Vietnamese paediatric tertiary referral hospital during a transition from blind biopsy to real‐time ultrasound‐guided biopsy. Models for performing real‐time ultrasound‐guided kidney biopsy differ across institutions. The procedure may be performed by nephrologists with ultrasound training or radiologists, depending on local expertise and workflow [1]. At our institution, implementation relied on a collaborative model in which the radiologist handled the ultrasound probe while the nephrologist advanced the biopsy needle. This model allowed real‐time visualisation while preserving nephrologist involvement in tissue acquisition, and may have supported the feasibility of transitioning to real‐time ultrasound guidance in our setting. Our findings show that this transition was associated with improved tissue yield, without a detectable increase in macroscopic haematuria or major complications. These data may be relevant to paediatric centres in similar resource‐constrained or transitioning settings that are seeking to improve biopsy adequacy while maintaining procedural safety.

It is well documented that both needle type (automated vs. manual) and diameter significantly affect glomerular yield [1]. Despite the variation in needle size and type, however, most studies concluded that real‐time ultrasound‐guided biopsy is superior to the blind technique, when performed with an automated biopsy gun [8–10, 21]. In contrast, a study conducted in adult patients using a 14‐gauge automated gun reported no difference between the two techniques, with all biopsies performed by attending nephrologists [22]. Similarly, another study reported higher glomerular yield with the blind Tru‐cut needle (14‐gauge) than real‐time ultrasound‐guided automated biopsy gun (18‐gauge) in adults [23], supporting evidence that larger needles improved yield [3]. In this study, the Tru‐cut procedures were performed by nephrologists, while the ultrasound‐guided biopsies were performed by other physicians. In contrast, a study, in which biopsies were performed by first‐year fellows under supervision, found that the blind Tru‐cut (14‐gauge) biopsies yielded fewer glomeruli than real‐time ultrasound‐guided automated biopsies (18‐gauge) [21]. These findings suggest that operator expertise can offset limitations of technique or equipment, with experience recognised as a major determinant of biopsy success, alongside the number of needle passes [21]. Few studies report data on the number of needle passes in a biopsy session, but both our study and Feneberg et al. showed that when the number of needle passes was comparable, real‐time ultrasound‐guided biopsy yielded significantly more glomeruli than the blind technique [10]. Overall, real‐time automated ultrasound‐guided biopsy generally provides superior glomerular yield, though skilled operators may achieve comparable results using manual techniques in the blind biopsy.

In our study, only one inadequate tissue sample was recorded, occurring in the blind biopsy group, with no significant difference between techniques. Similar findings were observed in several studies, in which biopsies were performed by nephrologists, including paediatric nephrologists [8, 10, 22]. In contrast, other studies found higher rates of inadequate sampling in blind biopsies performed by fellows under supervision [9, 21]. Additionally, in a study by Kim et al., the implementation of real‐time pathological assessment allowed termination once adequate tissue was obtained, resulting in no inadequate samples [23]. Collectively, these findings highlight operator experience and real‐time pathology involvement as key factors in minimising inadequate sampling.

A biopsy sample with ≥ 10 glomeruli is generally considered adequate for LM, while ≥ 25 glomeruli provide approximately 95% sensitivity for detecting focal lesions [1, 4, 5]. Few studies reported these specific metrics. In our study, real‐time ultrasound‐guided biopsy achieved higher proportions of samples with ≥ 10 and ≥ 25 glomeruli than the blind technique. Another study also demonstrated its superiority, though their counts included IF specimens [9]. Conversely, Feneberg et al. found similar proportions of samples with ≥ 10 glomeruli between groups despite higher total glomeruli with real‐time ultrasound‐guided biopsy [10]. This suggests that efficient sample division can preserve glomeruli for LM even with lower overall yield. Thus, both glomerular count and tissue sample division are critical for optimising diagnostic adequacy.

Post‐biopsy bleeding events in our study included macroscopic haematuria and one erythrocyte transfusion for symptomatic perinephric haematoma. Reported rates of macroscopic haematuria after kidney biopsy vary widely, from < 5% to as high as 40%–50% [1, 3]. Similar to other studies [9, 10, 22], we found no significant difference in macroscopic haematuria rates between real‐time ultrasound‐guided and blind biopsies. In contrast, a study reported higher macroscopic haematuria rates in blind biopsies [8], possibly related to more needle passes, as their target of three cores per first biopsy may have required additional attempts—an association noted previously [21].

Major complications were rare in our cohort. Only one patient, in the real‐time ultrasound‐guided biopsy group, required erythrocyte transfusion for symptomatic perinephric haematoma, and no cases required invasive intervention or surgery. No perinephric abscess or death was recorded. Similarly, several studies reported no significant differences in erythrocyte transfusion rates and no other major complications [8, 9, 22]. Because only one major bleeding event occurred, the study was not powered to evaluate predictors of major bleeding. Overall, our findings suggest that real‐time ultrasound‐guided biopsy improved tissue yield without a detectable increase in post‐biopsy bleeding events in this cohort.

Low eGFR was independently associated with post‐biopsy bleeding events, which were predominantly macroscopic haematuria. Each 10 mL/min/1.73 m^2^ decrease in eGFR was associated with approximately 1.24‐fold higher odds of post‐biopsy bleeding events (95% CI 1.03–1.49). Low eGFR may increase bleeding tendency through impaired platelet function, particularly in advanced kidney disease [24]. Although prior studies identified high needle pass numbers, low haemoglobin and hypertension as risk factors [1, 25], these were not significant in our cohort, likely due to strict pre‐biopsy screening and a policy of minimising needle passes. While routine post‐biopsy ultrasound is not generally recommended for detecting asymptomatic haematomas [1, 7], immediate and 6‐h post‐biopsy ultrasound has been suggested to help predict bleeding risk [26]. Therefore, careful pre‐biopsy evaluation and limiting needle passes remain important, particularly in patients with low eGFR.

### 4.1. Clinical Implications

Real‐time ultrasound‐guided kidney biopsy in children provides higher glomerular yield than the blind technique, without a detectable increase in macroscopic haematuria or major complications in this cohort. Lower eGFR was associated with post‐biopsy bleeding events, highlighting the importance of pre‐biopsy assessment and minimising needle passes in patients with a low eGFR.

### 4.2. Limitations

The study is limited by its single‐centre, retrospective design, lack of routine post‐biopsy ultrasound monitoring and absence of follow‐up haematocrit or haemoglobin measurements, which may have missed asymptomatic bleeding. Technique differences over time, including blind biopsies in earlier patients and real‐time ultrasound‐guided biopsies in later patients, and radiologist involvement in real‐time procedures may have influenced biopsy outcomes. In addition, because only one patient required erythrocyte transfusion, the study was not powered to evaluate predictors of major bleeding. Nonetheless, 7 years of continuous use of both techniques in our study provides meaningful comparative data.

## 5. Conclusion

Real‐time ultrasound‐guided biopsy demonstrates superior glomerular yield compared with the blind technique in paediatric patients, without a detectable increase in macroscopic haematuria or major complications. In a Vietnamese tertiary referral setting, implementation of real‐time ultrasound‐guided biopsy was feasible through nephrology‐radiology collaboration and improved tissue adequacy. Operator expertise, proper sample division and real‐time pathology review may minimise inadequate sampling. Low eGFR was associated with post‐biopsy bleeding events in this cohort, mostly macroscopic haematuria rather than major bleeding.

**Nomenclature**
AOR: Adjusted odds ratio
BMI: Body mass index
CCH: City Children’s Hospital
eGFR: estimated glomerular filtration rate
INR: international normalised ratio
IQR: interquartile range
RPGN: rapid progressive glomerulonephritis
SPSS: Statistical Package for the Social Sciences
SRNS: steroid‐resistant nephrotic syndrome
WHO: World Health Organization

## Author Contributions

Binh Thanh Le and Minh Cuong Duong designed the study. Binh Thanh Le performed data collection. Binh Thanh Le and Minh Cuong Duong performed statistical analysis and drafted the manuscript. All authors revised the paper critically for important intellectual content.

## Funding

The authors have nothing to report. Open access publishing facilitated by University of New South Wales, as part of the Wiley ‐ University of New South Wales agreement via the Council of Australasian University Librarians.

## Disclosure

All authors read and approved the final manuscript.

## Ethics Statement

This study received ethical approval from the Ethics Committees of City Children’s Hospital (approval No. 1010/QĐ‐BVNDTP).

## Conflicts of Interest

The authors declare no conflicts of interest.

## Data Availability

The de‐identified data and analysis code may be made available upon reasonable request to the corresponding author, pending approval from relevant institutional review boards and data custodians.
